# Toddlers infer unobserved causes for spontaneous events

**DOI:** 10.3389/fpsyg.2014.01496

**Published:** 2014-12-23

**Authors:** Paul Muentener, Laura Schulz

**Affiliations:** ^1^Department of Psychology, Tufts UniversityMedford, MA, USA; ^2^Department of Brain and Cognitive Sciences, Massachusetts Institute of TechnologyCambridge, MA, USA

**Keywords:** causation, prediction, intervention, exploration, toddler

## Abstract

Previous research suggests that children infer the presence of unobserved causes when objects appear to move spontaneously. Are such inferences limited to motion events or do children assume that unexplained physical events have causes more generally? Here we introduce an apparently spontaneous event and ask whether, even in the absence of spatiotemporal and co-variation cues linking the events, toddlers treat a plausible variable as a cause of the event. Toddlers (24 months) saw a toy that appeared to light up either spontaneously or after an experimenter’s action. Toddlers were also introduced to a button but were not shown any predictive relation between the button and the light. Across three different dependent measures of exploration, predictive looking (Study 1), prompted intervention (Study 2), and spontaneous exploration (Study 3), toddlers were more likely to represent the button as a cause of the light when the event appeared to occur spontaneously. In Study 4, we found that even in the absence of a plausible candidate cause, toddlers engaged in selective exploration when the light appeared to activate spontaneously. These results suggest that toddlers’ exploration is guided by the causal explanatory power of events.

## INTRODUCTION

Hume (1739/1978) famously claimed that we never actually see causal events. All we observe are a set of predictive relations. One billiard ball strikes another and the second one moves. A baseball player swings a bat at a baseball and the baseball soars over the outfield wall. However, the human mind represents these events as causal relations, and not only with respect to simple physical events but with relations that are harder to visualize: smoking *causes* lung cancer, the moon’s movement *causes* the tide to ebb and flow, and the baseball player *causes* the team to score a run. The ability to represent events causally shapes much of learning and development.

There have been multiple ways of looking at children’s causal inference, focusing variously on reasoning about spatiotemporal properties of causal events, the dispositional status of the entities involved in causal events, domain-specific theories about causal relationships, and domain-general abilities to integrate these theories with patterns of evidence. For example, researchers have tested infants’ sensitivity to spatiotemporal parameters (e.g., physical contact and temporal immediacy), showing that small changes in these events (spatial gaps and temporal delays) change infants’ percept from a causal event to a non-causal event (Leslie, 1982, 1984a; Leslie and Keeble, 1987; Oakes and Cohen, 1990; Cohen and Oakes, 1993; Oakes, 1994; Cohen and Amsel, 1998; Kotovsky and Baillargeon, 2000; Belanger and Desrochers, 2001; Newman et al., 2008). Other research has looked at children’s intuitive theories of the physical, psychological, and biological worlds (Carey, 1985; Spelke et al., 1992; Wellman and Gelman, 1992; Inagaki and Hatano, 1993; Kalish, 1996; Gopnik and Wellman, 2012), showing that children have domain-specific expectations for physical and psychological causality. Infants are also sensitive to the ontological status of participants in causal events, changing their representations depending on whether the entities are objects or agents (Leslie, 1984b; Woodward et al., 1993; Kotovsky and Baillargeon, 2000; Kosugi and Fujita, 2002; Kosugi et al., 2003; Saxe et al., 2005, 2007; Luo et al., 2009; Muentener and Carey, 2010) Finally, many researchers have looked at how children’s folk theories are integrated with patterns of data to support prediction, intervention, explanation, and counterfactual reasoning across domains (Gopnik et al., 2004; Schulz and Gopnik, 2004; Kushnir and Gopnik, 2007; Schulz et al., 2007; Sobel and Munro, 2009; Bonawitz et al., 2010, 2012; Kushnir et al., 2010; Legare, 2012).

Underlying all these questions, however, is the assumption that humans come to treat the world in terms of causal relationships, including unobserved causal relationships, rather than simply as sets of observable events. The abstract expectation that all events have causes may be a core feature of human cognition, emerging very early in development. In the current paper, we look at whether toddlers represent events as having plausible candidate causes *in the absence of* any spatiotemporal cues or data itself indicative of a causal relationship. In particular, we ask whether toddlers believe that unexplained, seemingly spontaneous physical events have causes.

Prior research suggests that by the age of five, children do tend to assume that spontaneously occurring physical events have causes. In classic research on causal reasoning, researchers showed that 5-year-olds denied that a physical outcome could occur spontaneously (Bullock et al., 1982). When asked to explain a novel, apparently spontaneous jack-in-the-box event, no child suggested that the event occurred on its own. Rather, all children referred to hidden variables (e.g., wires, remote controls, or “invisible batteries”). Similarly, researchers showed that 5-year-olds posited hidden variables when causes appeared to act probabilistically (Schulz and Sommerville, 2006).

Research conducted on younger children, however, presents a more complex picture. Much of the research on infants’ intuitive theories about objects’ physical interactions and agents’ goal-directed actions suggests that they may believe that spontaneous events have causes. For example, 5- to 6.5-month-old infants infer that a box that moves without contact by a human hand can also switch direction, resist force, and change location (Luo et al., 2009). The researchers suggest that infants in these studies infer that the box is a ‘self-propelled agent,’ and that its motion is internally caused. Similarly, researchers have shown that infants will treat inanimate objects as if they are engaging in goal-directed behavior if the objects can spontaneously alter their direction of motion (Luo and Baillargeon, 2005; Biro and Leslie, 2007; Johnson et al., 2007). Such studies suggest that infants categorize entities as either agents or objects depending on their pattern of behavior, including the likelihood of spontaneous movement and spontaneous changes in trajectory. However, these studies do not specifically ask infants to reason about the causes of apparently spontaneous behavior. Rather, these studies focus on whether infants believe that some properties of agents predict other properties of agents (e.g., if something moves without external force, should it also be able to change direction). Infants might have domain-specific cues to agency along with a set of domain-specific inferences about agents, but still fail to posit causes for apparently spontaneous events in objects.

Saxe et al. (2005,^2007^) provide stronger evidence that infants infer causes for apparently spontaneous physical events. If a beanbag emerges in motion, infants, by around 10 months of age, seem to infer that an unseen agent generated the movement. Infants are less surprised if a hand emerges at the origin of the motion trajectory than at its terminus, and this inference only holds for agents (plausible candidate causes of the motion), not for objects (e.g., toy trains). That is, infants seem both to resist the idea that the object can move spontaneously and to consider the possibility of hidden causes.

These studies suggest that infants expect agents to initiate motion events. However, we do not know to what extent such inferences extend beyond motion events. Moreover, infants might find the appearance of one candidate cause more plausible than the other (the hand at the appropriate location versus the wrong location) without necessarily positing the existence of a cause in the first place. Indeed if infants require observed covariation data, contact causality, or observed interventions for causal inference (see e.g., Rescorla and Wagner, 1972; Leslie and Keeble, 1987; Gopnik et al., 2004), then there is no reason to suppose that the mere appearance of a spontaneous event should suffice for them to infer causal relationships. By contrast, if infants assume that apparently spontaneous events have causes, we might expect them to (1) look predictively between the activation of the candidate cause and the outcome and (2) selectively intervene on candidate causes given otherwise unexplained events. We test these two predictions in the current study. Because our measures extend beyond looking time, here we test toddlers (24 months) rather than infants.

In the current study, we introduce toddlers to a causal event: a toy box that lights up either when contacted by the experimenter (Observed condition) or spontaneously (Spontaneous condition). We then reveal a previously hidden candidate cause: a button physically connected to the box. Toddlers’ prior knowledge is consistent with the possibility that the button might activate the light: buttons are a plausible cause of the light’s activation much as hands are a plausible cause of the beanbag’s motion. However, toddlers never see the button associated with the light activation. We predict that toddlers will treat the button differently depending on whether the light apparently turned on after the experimenter’s intervention or spontaneously. In the Observed condition, we predict that toddlers will neither look predictively from the button to the light, nor selectively intervene on the button; the observed intervention provides a plausible cause for the event. By contrast, if toddlers resist spontaneous events and assume that there was an unobserved cause of the light’s activation, then in the Spontaneous condition, toddlers should both predictively look toward the light when the button is pushed, and selectively intervene on the button.

Importantly, while toddlers view the spontaneous causal event, no goal-directed action immediately precedes the events occurrence, the button is occluded from the child’s view, and no other event predicts the toy lighting up. Thus, although the button is a plausible candidate cause with respect to toddlers’ domain-specific prior knowledge, toddlers are given neither spatiotemporal cues connecting the button to the light, nor conditional probability evidence for a causal relationship between the button and the light. If toddlers always connect plausible causes with their outcomes, they should do so both in the Observed and Spontaneous conditions; however, if toddlers selectively search for plausible causes given otherwise unexplained events, they should be more likely to connect the cause with the light (in both prediction and action) in the Spontaneous condition.

In the current study, we use three different measures to assess toddlers’ causal inferences. In Experiment 1, we ask whether toddlers infer a predictive relation between the button and the outcome only when it occurs spontaneously by measuring their predictive looking following an action on the button. In Experiment 2, we prompt toddlers to turn on the light and assess whether they press the button. In Experiment 3, we do not prompt toddlers and assess whether they spontaneously attempt to cause the light to activate by pressing the button. Finally, in Experiment 4, we look at whether toddlers search for candidate causes of spontaneous physical events even when no plausible mechanism such as a button is available.

## EXPERIMENT 1

In Experiment 1 we use a predictive looking paradigm to see whether toddlers selectively connect plausible candidate causes with otherwise apparently spontaneously occurring events. Toddlers are introduced to a novel toy box and shown that a light on the box flashes on. Half the toddlers are given a plausible cause of the light’s activation: the experimenter touches the box and the light turns on (Observed condition); research suggests that even infants treat intentional contact by a human hand as a plausible cause for an artifact changing state (Muentener and Carey, 2010). The remaining toddlers are not given a plausible cause and thus the light appears to activate spontaneously (Spontaneous condition). The light is then occluded and all toddlers see the experimenter press a button connected to the box. Thus, the toddlers do not receive any evidence that the button press covaries with the light’s activation. During test trials, the occluder is removed and the experimenter presses the button; the light never activates during the test trial. We code whether toddlers look toward the toy after the experimenter presses the button.

Whether or not toddlers expect unexplained events to have causes, they might expect buttons to activate lights. If so they might look from the button to the light box in the Observed condition as well as the Spontaneous condition. Indeed because the light box is the only other artifact on the stage, they might look to the light box even at baseline. Thus we also include a Button Control condition in which the experimenter presses the button but the light never activates. If toddlers look from the button to the box, not because they are looking for a cause of the effect, but because they are looking for an effect for the cause – or simply because the box itself attracts their attention – then they should also look toward the box in the Button Control condition. However, if toddlers selectively posit causes of otherwise unexplained event, toddlers should look toward the box following the button press only in the Spontaneous condition.

### MATERIALS AND METHODS

#### Participants

Forty-eight toddlers (mean: 25.08 months, range – 18–30 months; 15 female) were recruited at a children’s museum. An additional 12 toddlers were recruited but not included in the final sample due to: inability to complete the session (*n* = 4), inattention (*n* = 1), parental interference (*n* = 4), or experimenter error (*n* = 3). Toddlers were assigned to the Observed condition, the Spontaneous condition, or the Button Control condition (*n* = 16/condition). There were no age differences between the conditions (*p* = ns).

#### Materials

The light box was constructed from a black box (6 inch × 6 inch × 6 inch) with a small blue lamp (2 in diameter) emerging from the front panel. The experimenter could control the light surreptitiously by pressing a button that was out of toddlers’ view. The button box was a small red button on top of an orange cylindrical case (4 inch diameter × 2 in high). Since physical contact between candidate causes and outcomes facilitates causal reasoning in both infant (e.g., Cohen and Amsel, 1998) and children’s (Kushnir and Gopnik, 2007) causal reasoning, we connected the button box to the light box with a long orange rod (15 inch). A black screen served as an occluder throughout the procedure. An additional black screen was placed behind the light box to obscure the experimenter’s surreptitious activation of the blue lamp.

#### Procedure

**FIGURE 1:**
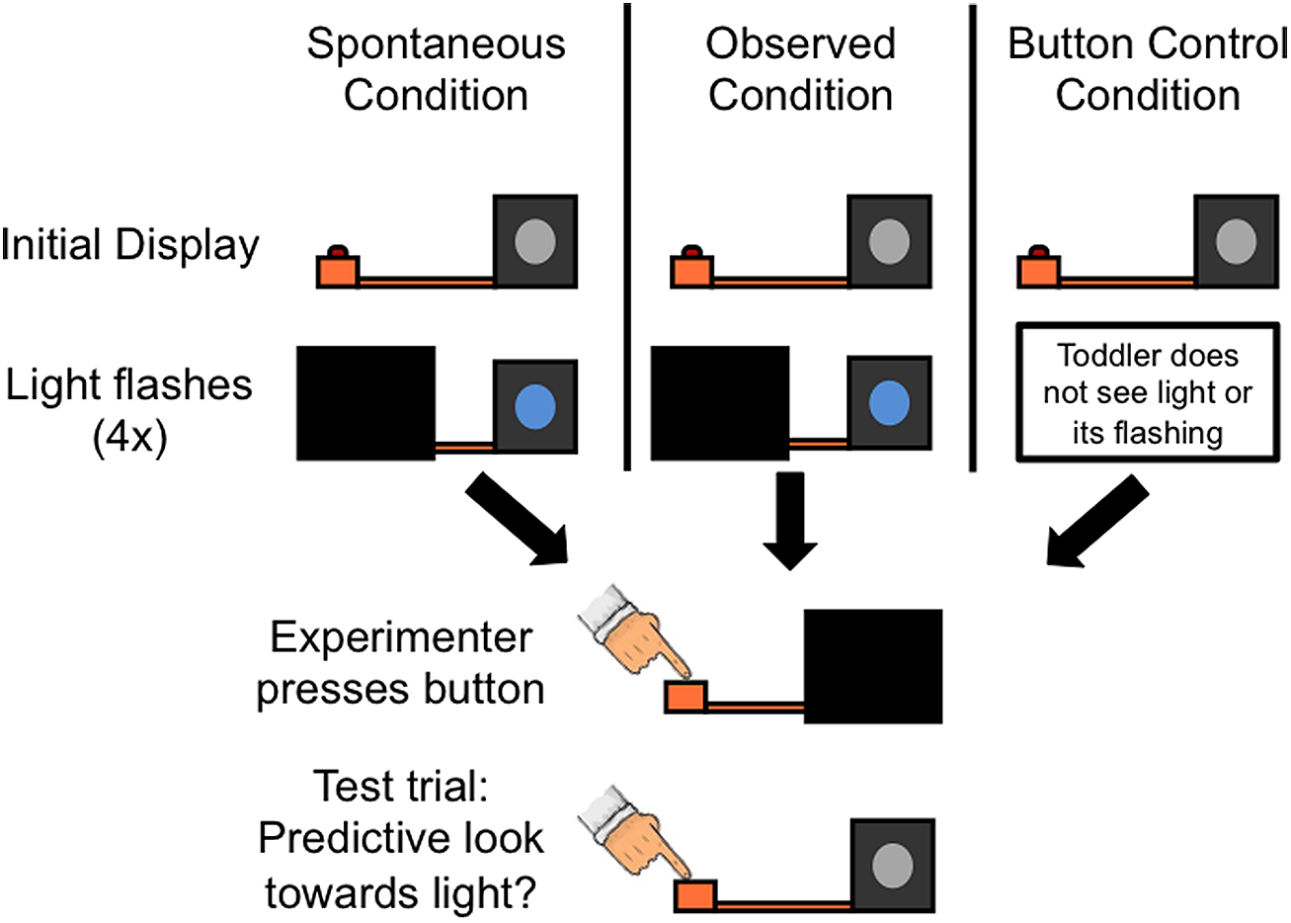
**Procedure for Experiment 1.** In the Spontaneous condition, toddlers first saw a button box connected to a black box with a blue disk on its front by an orange rod (Initial display). The experimenter then occluded the button box and surreptitiously made a light on the front of the box flash (Light flashes). The Observed condition differed from the Spontaneous condition only in that the experimenter touched the light box immediately prior to it flashing. Although it appeared that the experimenter activated the light box via contact, she actually still controlled the light box in the same manner as the Spontaneous condition. In the Button Control condition, toddlers did not see to what the button box was connected (i.e., the light box). They also did not see the light flash. Next, in all three conditions, the experimenter occluded the light box and pressed the button. Finally, the experimenter revealed the entire display and pressed the button again. Note that although the light appears to remain ‘on’ in the figure, the light in fact flashed on and off four times before ending in the off state before proceeding to the next phase of the experiment. Note also that in all conditions the experimenter sat behind the stimuli, directly across from the child, such that from the child’s perspective she could have contacted both the button box and the light box throughout the experiment.

All procedures were approved by the MIT Institutional Review Board. Toddlers were tested individually in a private testing room. The entire experimental session was videotaped for subsequent coding by an individual, blind to condition. The camera was placed to the side of the experimenter, facing the child, such that the direction of the child’s gaze could be easily coded. In the Observed and Spontaneous conditions, the stimuli were sitting on a table when the child entered the room. The experimenter sat behind the stimuli, facing the child; she placed one hand behind the light box and black screen. The experimenter directed the child’s attention to all components of the stimuli (the button, the connected rod, and the light box), without labeling the specific items (e.g., “Look at this”; **Figure 1**). The experimenter then placed the occluder in front of the button box to obscure toddlers’ view. Upon placing the occluder in front of the button box, the experimenter inconspicuously rested her other hand behind the occluder. Thus, although the experimenter did not engage in any overtly intentional actions behind the button screen from the child’s perspective, the experimenter had access to all parts of the stimuli and could plausibly have pushed the button in both conditions.

In the Observed condition, the experimenter touched the rim of the light and then the box lit up and flashed blue (four rapid flashes on and off in quick succession, approximately 1 s total). In the Spontaneous condition, the experimenter did not touch the box and toddlers saw the box light up and flash blue apparently spontaneously. The light was off at the end of this phase of the experiment. In both conditions the experimenter then moved the occluder to reveal the button box and occlude the light box. The experimenter pushed the button for 1 s in both conditions.

The procedure was similar for toddlers in the Button Control condition, except that when the toddlers walked into the testing room, the light box was occluded and only the button box and the connecting rod were visible. The experimenter directed the toddlers’ attention to the button without labeling it (“Look at this”) and pressed the button. Therefore, the only difference between this condition and the Spontaneous and Observed conditions was the absence of an introduction to the light box at the start of the experiment and the display of the light flashing.

During the test trial in all conditions, the experimenter removed the occluder so that all components were visible to the child. The experimenter pressed the button but the light box did not activate. Most toddlers spontaneously attended to the experimenter and her actions throughout the experiment; if toddlers were not attending to the experimenter at the start of the test trial, the experimenter would use non-specific attentional statements (“Look at this”) to re-direct the child’s attention. Thus, all toddlers were looking at the button when the experimenter pressed button. Following prior research using predictive looking measures (Bonawitz et al., 2010; Muentener et al., 2012a,b) a coder, blind to condition, assessed whether toddlers’ first look in the 3-s window following the button press was to the light box (which was off). A second coder, also blind to condition, recoded 33% of the data. Inter-coder reliability was high (κ > 0.8).

#### Results and Discussion

**FIGURE 2:**
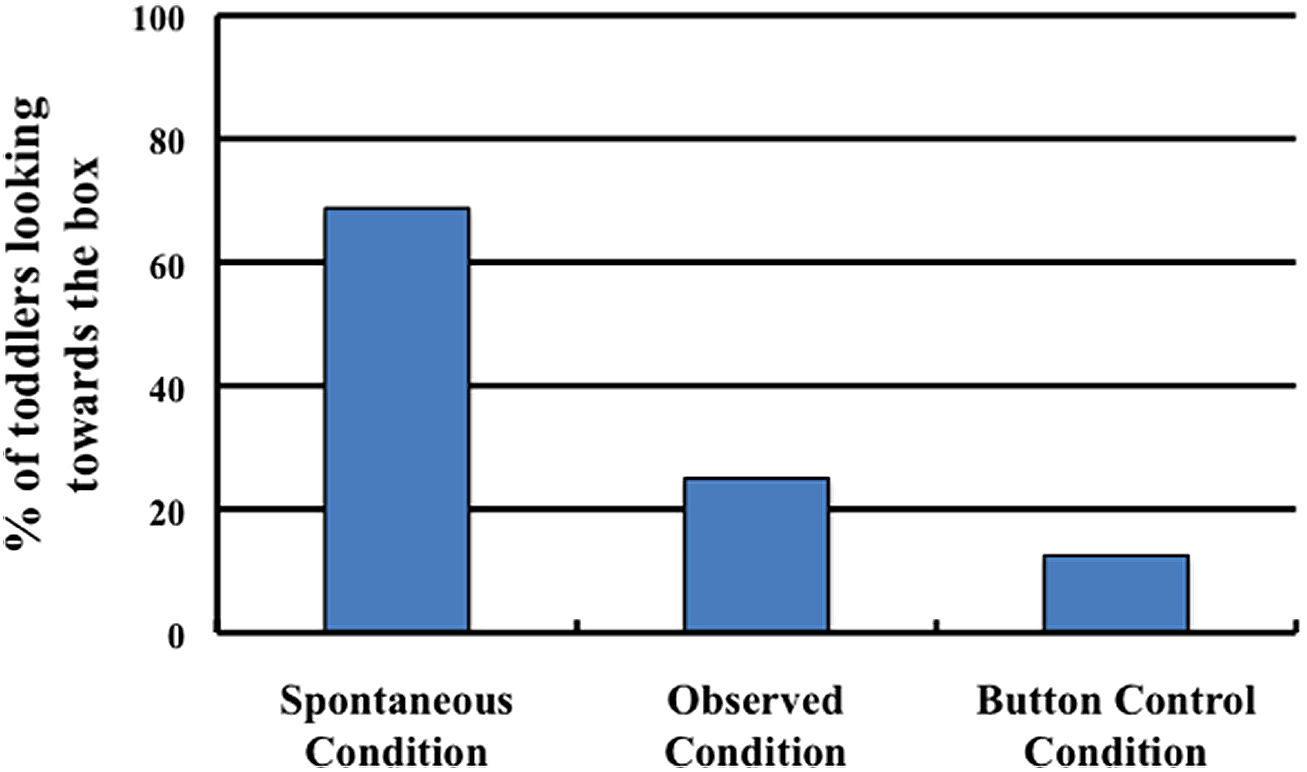
**The percentage of toddlers in each condition of Experiment 1 who looked toward the light box after the experimenter pressed the button during the test trial**.

Our primary measure of interest was whether toddlers looked toward the light box (which was inert during the test trial) following the experimenter pressing the button. As predicted, the toddlers were significantly more likely to look to the box in the Spontaneous condition (68.75%, 11/16 toddlers) than in either the Observed condition (25.00%, 4/16 toddlers; Fisher’s exact test, *p* < 0.05) or the Button Control condition (12.50%, 2/16 toddlers; Fisher’s exact test, *p* < 0.05; **Figure 2**). There was no significant difference in the number of toddlers’ that looked toward the light box in the Observed and Button Control conditions (*p* = *ns*).

These results are consistent with the possibility that 2-year-olds believe that unexplained physical events have causes. Even though they had never seen any evidence that the button press and the light co-varied, toddlers looked from the button press to the light when the light had apparently occurred spontaneously. Toddlers did not look from the button press to the light box when the light could be explained by the experimenter’s action, nor did they look at the light box simply in response to the button press. They only did so when they had previously seen an otherwise unexplained event.

The results of Experiment 1 suggest that toddlers inferred a relationship between the novel event and the candidate cause only when there was an event to explain and that event had no other candidate explanation. However, they do not establish that the toddlers believed the button press actually *caused* the light to activate. Additionally, the experimenter touched the light box in the Observed condition but not in the Spontaneous condition. Arguably the experimenter’s contact with the light box in the Observed condition might have shifted the toddlers’ attention away from the button.

In Experiment 2, we conducted a stronger test of toddlers’ inference that unexplained physical events have causes. We looked at whether toddlers selectively intervene on the button in the Spontaneous condition relative to the Observed condition. We also addressed the concern about differential contact with the light box. In Experiment 2, the experimenter touched the light box in both conditions: either immediately after the light activated (Spontaneous condition) or immediately before (Observed condition).

## EXPERIMENT 2

### MATERIALS AND METHODS

#### Participants

Thirty-two toddlers (mean: 24.96 months, range – 18–30 months, 17 female) were recruited at a children’s museum. Seven additional toddlers were recruited but not included in the final sample due parental interference (*n* = 3) and failure to intervene (*n* = 4). Toddlers were assigned to either the Spontaneous or Observed condition (*n* = 16/condition). There were no age differences between conditions (*p* > 0.05).

#### Materials

The same materials used in Experiment 1 were used in Experiment 2.

#### Procedure

**FIGURE 3:**
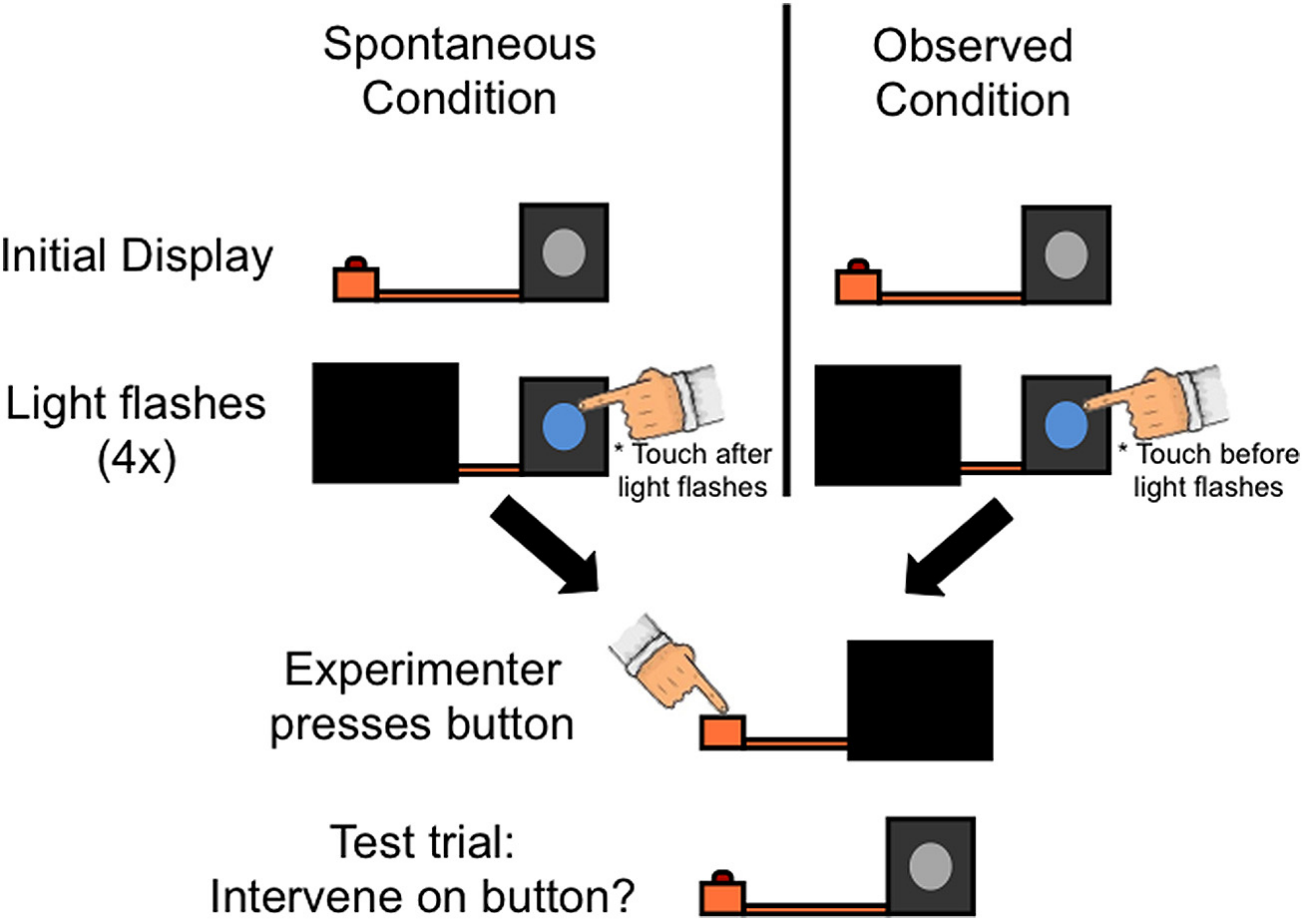
**Procedure for Experiment 2.** All infants first saw that a button was connected to a box by an orange rod. In the Spontaneous condition, the experimenter then occluded the button and surreptitiously made a light on the front of the box flash (Light flashes); immediately after the light flashed on and off, the experimenter touched the light. The Observed condition differed from the Spontaneous condition only in that the experimenter touched the light immediately prior to it flashing. As in Experiment 1, although it appeared that the experimenter activated the light via contact, she actually still controlled the light in the same manner as the Spontaneous condition. Note that although the light appears to remain ‘on’ in the figure, the light in fact flashed on and off four times before ending in the off state before proceeding to the next phase of the experiment. Next, in both conditions, the experimenter occluded the light box and pressed the button. Finally, the experimenter revealed the entire display and asked the toddler to “make the light turn on.”

The procedure was similar to Experiment 1 with the following changes (see **Figure 3**). The experimenter touched the light box in both conditions: in the Observed condition, the experimenter touched the light box immediately before the light activated (as in Experiment 1); in the Spontaneous condition the experimenter touched the light box immediately after the light turned on (so that it looked like a response to, rather than potential cause of, the light activating).

During the test event the experimenter did not push the button. Instead, the experimenter pushed the stimuli toward the child and asked the child to “make the light turn on.” Toddlers were given 30 s to interact freely with the button and light box apparatus.

A coder, blind to condition, recorded toddlers’ actions and looking behavior. Our primary measure of interest was whether toddlers intervened on the button within a 30-s window following the prompt. If a child touched the button in the 30-s window, we also coded whether the child’s first look after touching the button was toward the light (which was inert). Since toddlers’ attention was usually directed at their actions, we could not assess toddlers’ predictive looks if their first action was touching the light box. Thus, we do not include any data assessing that behavior in this experiment.

A second coder, also blind to condition, recoded 33% of the data. Inter-coder reliability was high (κ > 0.8).

#### Results and Discussion

**FIGURE 4:**
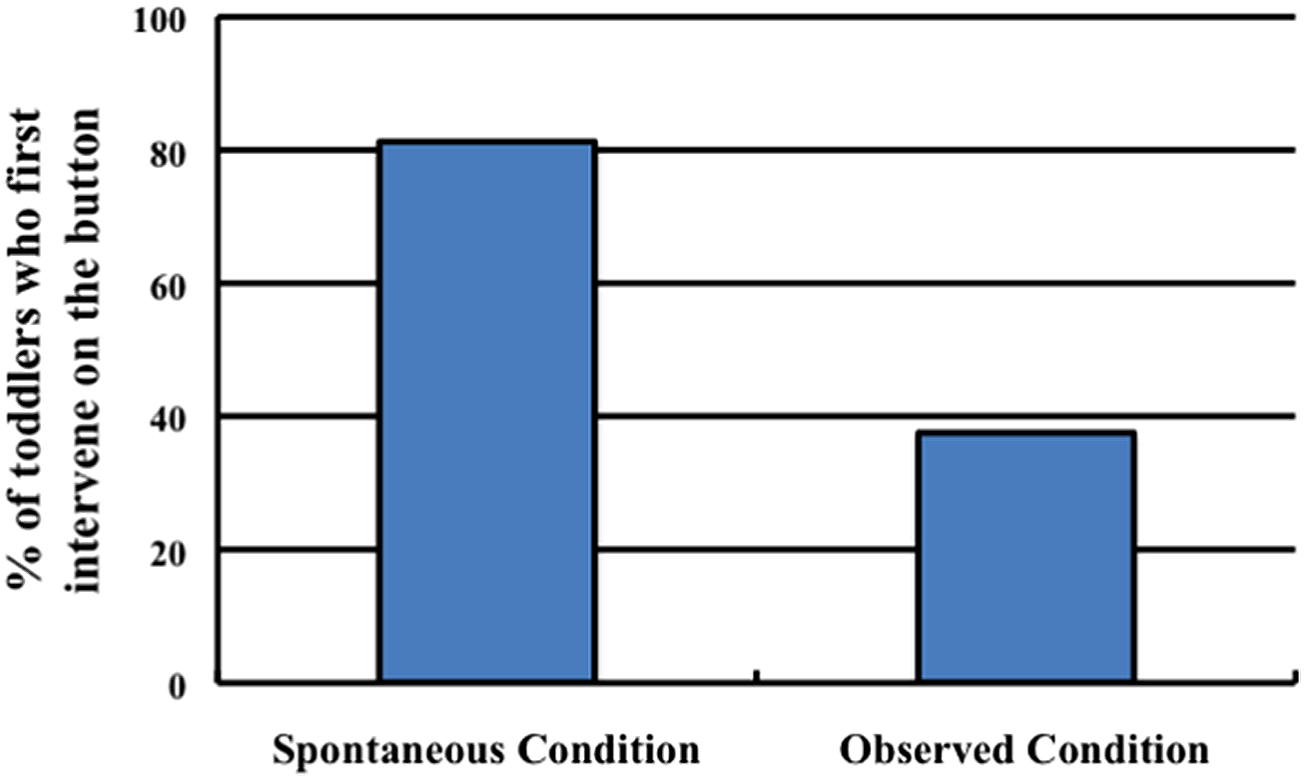
**The percentage of toddlers in each condition of Experiment 2 whose first action on the stimuli was to contact the button**.

Our primary measure of interest was whether toddlers’ first action on the stimuli was directed toward the button. All toddlers directed their first action toward either the button or the light box. Analysis of toddlers’ actions revealed that they were more likely to push the button in the Spontaneous condition (81.25%, 13/16 toddlers) than in the Observed condition (37.50%, 6/16 toddlers; Fisher’s Exact test, *p* < 0.05; see **Figure 4**). In contrast, toddlers were more likely to touch the box in the Observed condition (62.50%, 10/16 toddlers) than in the Spontaneous condition (18.75%, 3/16 toddlers). These results suggest that toddlers were more likely to posit the button as a cause of the light’s activation when they did not have an existing explanation.

It is also important to note that the experimenter contacted both the button and the light in both the Spontaneous condition and Observed conditions. The only difference between the conditions was whether the experimenter’s action on the light could be represented as a cause of the lights flashing; in the Observed condition it could, but in the Spontaneous condition it could not. Thus, the toddlers differentially imitated the experimenter’s actions depending on their causal attributions.

Taken together Experiments 1 and 2 suggest that toddlers expect spontaneously occurring events to have a cause, and that this belief guides their visual and manual search. However, in both experiments, toddlers were given the potentially relevant causal action: pressing a button. We do not know whether toddlers in the Spontaneous condition (1) inferred the presence of an external cause and actively searched for it or (2) whether they linked the two subevents of the spontaneous light flash and the button press only *after* the experimenter directed the child’s attention toward the button by pressing it. Additionally, toddlers were prompted to “make the light turn on” during the test event, which may have additionally cued to the child that there was a cause to the light activation in the Spontaneous condition, even though they may not have made such an inference prior to the prompt. If toddlers believe strongly that unexplained events have causes, then toddlers might also search for a candidate cause even if the experimenter does not direct the toddlers’ attention toward the cause and directs them to explore. We test this prediction in Experiment 3. Since in this experiment, toddlers will not see any intervention on the button, we look both at toddlers’ first actions and their overall pattern of exploration across conditions.

## EXPERIMENT 3

### MATERIALS AND METHODS

#### Participants

Thirty two toddlers (mean: 23.16 months, range – 18–30 months; 14 female) were recruited at a children’s museum. Thirteen additional toddlers were recruited but not included in the final sample due to an inability to complete the session (*n* = 1), parental interference (*n* = 4), and failure to interact with the stimuli (*n* = 8). Toddlers were assigned to either the Observed or Spontaneous condition (*n* = 16/condition). There were no age differences between conditions (*p* = *ns*).

#### Materials

The same materials used in Experiment 1 were used in Experiment 3.

#### Procedure

**FIGURE 5:**
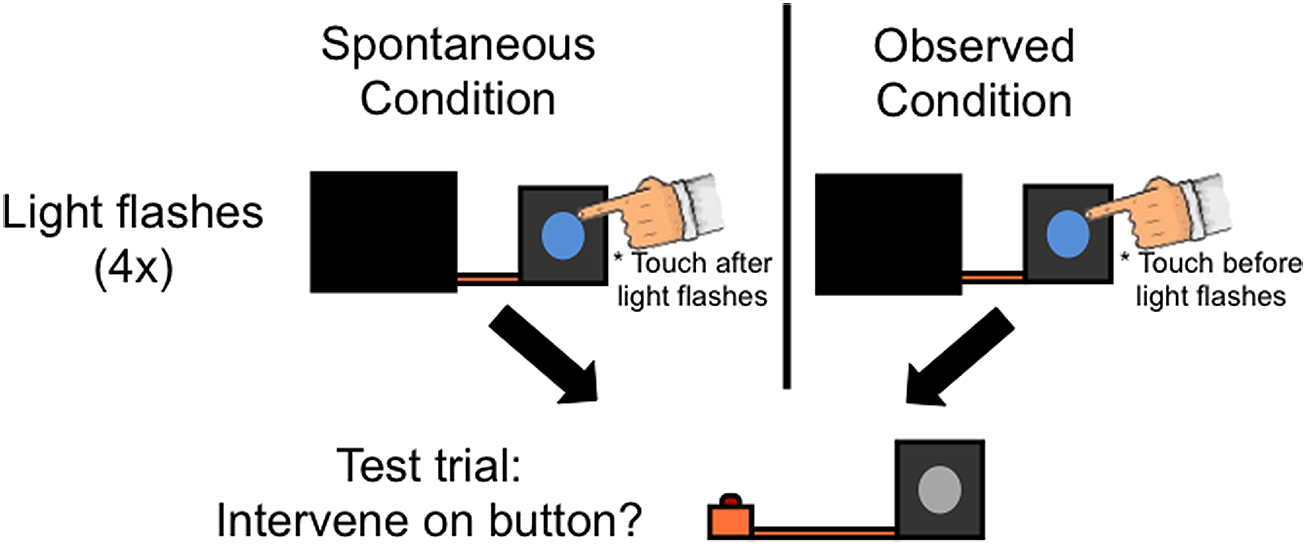
**Procedure for Experiments 3.** The procedure mirrored Experiment 2, except that the toddlers never saw the button connected to the box, and did not see the experimenter press the button. During the test trial the experimenter revealed the entire display and told toddlers that it was there “turn to play.”

The procedure was similar to Experiment 2 except that the toddler did not see the button until the test event (see **Figure 5**). When the child entered the testing room, the button was already occluded from the child’s view. Only after the toddler watched the light activate either as a result of the Experimenter’s contact (Observed condition) or spontaneously (Spontaneous condition) did the Experimenter remove the screen from in front of the button. The Experimenter then told the child it was his/her turn to play. She did not make any reference to the button and did not ask the child to turn on the light.

A coder, blind to condition, assessed whether toddlers intervened on the button within the first 30 s of interaction with the stimuli. The coder also coded where toddlers looked following their first action on the button. A second coder, also blind to condition, recoded 33% of the data. Inter-coder reliability was high (κ > 0.8).

#### Results and Discussion

**FIGURE 6:**
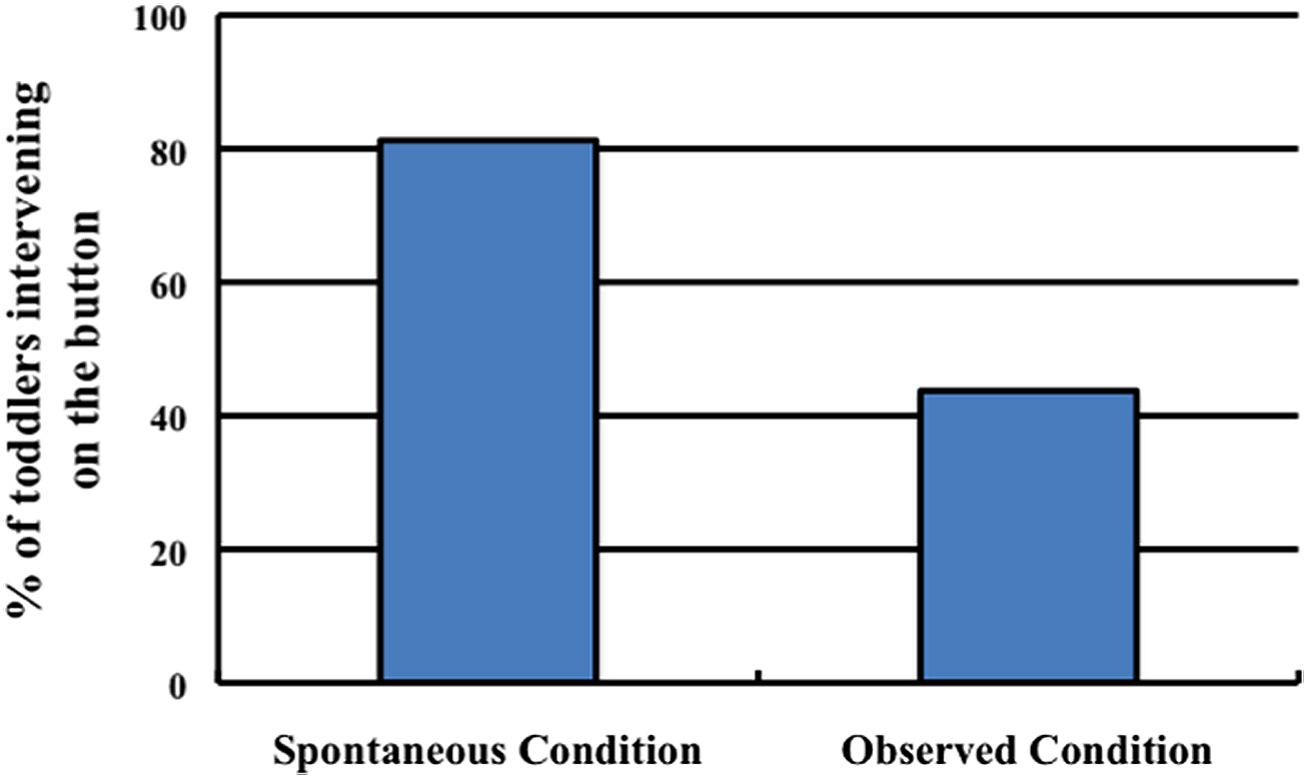
**The percentage of toddlers in each condition of Experiment 3 who contacted the button during the 30-s test trial**.

As expected, the majority of toddlers in both conditions (Observed condition: 75.00%, 12/16 toddlers; Spontaneous condition: 68.75%, 11/16 toddlers) directed their first action toward the light box. However, toddlers in both conditions continued to explore over the 30-s test trial. During the remainder of the test trial, toddlers were also more likely to press the button in the Spontaneous condition (81.25%, 13/16 toddlers) than in the Observed condition (37.50%, 6/16 toddlers; Fisher’s Exact test, *p* < 0.05; see **Figure 6**). Thus even though toddlers had never seen evidence that the button and the light covaried, had never seen the experimenter push the button, and were not prompted to turn on the light, toddlers selectively explored the button when the light seemed to activate spontaneously.

One concern with the previous experiments is that the condition differences may reflect reduced exploration of the button in the Observed condition rather than selective exploration in the Spontaneous condition. That is, toddlers may be more engaged by the light box in the Observed conditions rather than more likely to posit causes in the Spontaneous conditions. In Experiment 4 we address this concern by removing the button altogether. Instead, we compare toddlers’ exploration of the light box and a novel distractor toy that is distinct in shape and color from the light box, and physically unconnected to the light toy. If our results reflect only selective engagement with the light box in the Observed condition, then toddlers should selectively explore the light box here as well and choose not to explore the novel toy. If instead, as we have hypothesized, toddlers selectively search for candidate causes in the Spontaneous condition, then in the absence of a plausible external cause such as the button, toddlers should selectively explore the light box in the Spontaneous condition. In contrast, although toddlers in the Observed condition should imitate the Experimenter’s action to turn on the light (i.e., by touching the lamp), after toddlers learn that the lamp does not turn on, they should selectively explore the novel toy.

These predictions, however, require a caveat. Whether a learner actually engages in search depends on many factors, including the learner’s prior knowledge, the size of the search space, and exploration/exploitation trade-offs relating the cost and benefit of exploration to the cost and benefit of other actions the learner might take (e.g., Gittens, 1979). If learners believe that spontaneous events have causes, they should posit the existence of unobserved causes for otherwise unexplained events; however, this does not mean that learners should necessarily *search* for such causes. Even as adults, we see events every day that we cannot explain; we may assume that these events have causes but we rarely bother to seek out the causes ourselves. In the absence of a well-constrained search space, the costs of such a search could easily outweigh the potential for information gain. Nonetheless, if toddlers actively search for plausible candidate causes when events appear to occur spontaneously, then they should be more likely to explore the light box in the Spontaneous condition than the Observed condition. We test this prediction in Experiment 4.

## EXPERIMENT 4

### MATERIALS AND METHODS

#### Participants

Thirty two toddlers (mean: 22.92 months, range – 18–30 months; 14 female) were recruited at a children’s museum. Two additional toddlers were recruited but not included in the final sample due to an inability to complete the session (*n* = 1) and experimenter error (*n* = 1). Toddlers were assigned to either the Spontaneous or Observed condition (*n* = 16/condition). There were no age differences between conditions (*p* > 0.05).

#### Materials

**FIGURE 7:**
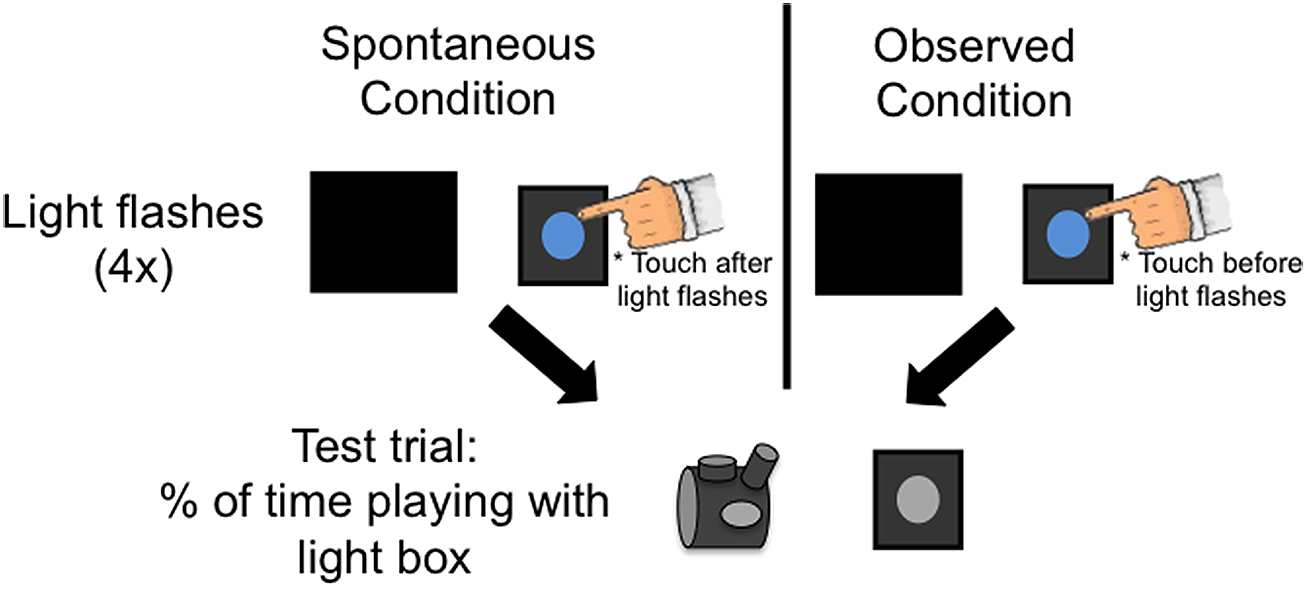
**Procedure for Experiments 4.** The procedure mirrored Experiment 3, except that the experimenter removed the occluder (which obscured the button box in Experiments 1–3) to reveal a novel, distractor toy. During the test trial the experimenter told toddlers that it was their “turn to play.”

The black screen and the light box from Experiments 1–3 were both used; however, the orange button box and connecting rod were removed. An additional custom-built toy (8 inch × 6 inch) served as the novel, distractor toy (**Figure 7**).

#### Procedure

Upon entering the testing space, toddlers saw the light box and the black screen (**Figure 7**). The screen occluded the distractor toy from the toddler’s view. After the toddler saw the novel event occur either spontaneously (Spontaneous condition) or as a result of the Experimenter’s contact (Observed condition), the Experimenter removed the screen from in front of the distractor toy, and then told the child it was his/her turn to play. She did not make any reference to the distractor toy and did not explicitly request that the child turn on the light. A coder, blind to condition, coded the amount of time the child played with the light box and with the distractor toy in the 30-s window following the removal of the screen. An additional coder, blind to condition, recoded 100% of the data. Inter-coder reliability was high (*r*^2^ > 0.9).

#### Results and Discussion

**FIGURE 8:**
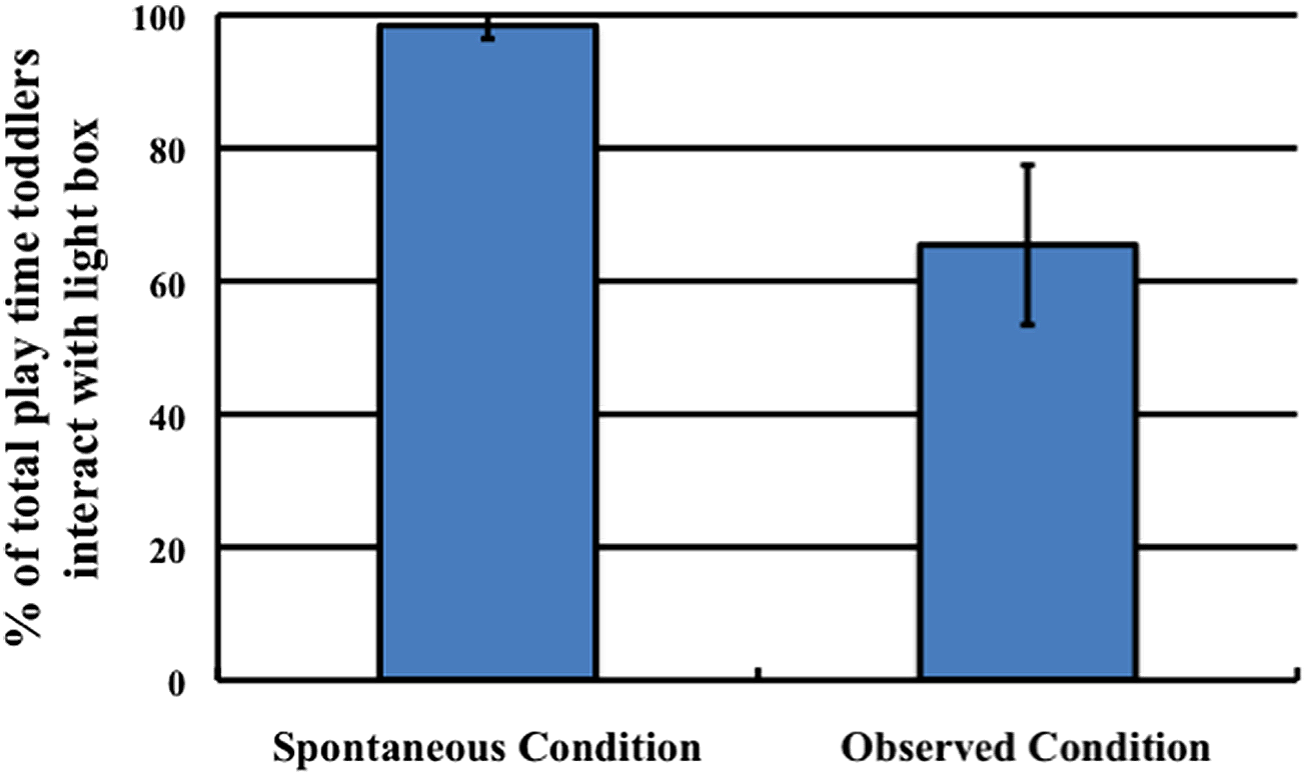
**The percentage of toddlers’ total play time of 30 s that they interacted with the light box, separated by condition.** Error bars denote ±1 SD.

In contrast to Experiments 2 and 3, toddlers were marginally more likely to refuse to interact with either toy in the Spontaneous condition (9/16 toddlers, 56.00%) than the Observed condition (3/16 toddlers, 18.75%; Fisher’s Exact test, *p* = 0.06). However, as predicted, when toddlers did play with the toys, they spent a greater percentage of their total playtime exploring the light box in the Spontaneous condition (98.41%) than in the Observed condition (65.43%), *t*(18) = 2.06, *p* = 0.05 (**Figure 8**).

These results should be interpreted with caution, as toddlers were less likely to play with the toys in these conditions, relative to the prior experiments, resulting in a small sample size for our analyses. However, they are consistent with the possibility that toddlers posit candidate causes when events appear to occur spontaneously. As importantly, they are inconsistent with the possibility that toddlers selectively attended to the light box in the Observed condition. Although the toddlers spent more than half their time playing with the light box in both conditions, toddlers in the Observed condition also explored the novel toy. By contrast, toddlers in the Spontaneous condition explored the light box almost exclusively.

Potentially the fact that the light box did not activate during the test period was especially discouraging to toddlers in the Observed condition, who had reason to expect that contact would activate the box. Note however, that the light box also failed to activate when toddlers intervened on it in Experiment 3. Nonetheless, in Experiment 3 toddlers were relatively more likely to direct attention away from the light box (to the button) in the Spontaneous condition whereas in Experiment 4 toddlers were more likely to direct attention away from the light box (to the novel toy) in the Observed condition. The overall pattern of results suggests that toddlers’ differential behavior in the two conditions was driven, not by variable attention to, or avoidance of the light box in the Observed condition, but by a selective search for a candidate cause when the events occurred spontaneously.

## GENERAL DISCUSSION

The results from the current study suggest that when toddlers see a novel physical event, they are more likely to predict relationships between a candidate cause and the event (Experiment 1) and intervene on and explore plausible candidate causes (Experiment 2 and Experiment 3) if the event appears to occur spontaneously than if they have an explanation for the event. Even when no plausible candidate cause is provided, toddlers engage in more exploration of the stimuli related to the event when the event appears to occur spontaneously than when they have an explanation for its occurrence (Experiment 4). Thus 2-year-olds appear to infer that physical events have causes, and both accept and search for plausible candidate causes of events, even in the absence of covariation cues linking candidate causes and effects.

Prior research with older children (Bullock et al., 1982; Schulz and Sommerville, 2006) had shown that children believe that physical events have causes by at least 5 years of age. Research also suggested that this belief might be present, at least for caused motion events, in younger infants (e.g., Saxe et al., 2005; Luo et al., 2009). The current study extends previous research beyond the case of caused motion and provides convergent evidence by using predictive looking, intervention, and exploration as measures of causal inference. Future research might investigate the developmental origins of this belief in infancy. Just as infants may have innate domain-specific knowledge about objects and their physical interactions and agents and their goal-directed actions (Spelke and Kinzler, 2007; Carey, 2009), the belief that all events have causes may be part of infants’ core causal knowledge, integrating information across domains and guiding the development of intuitive theories in early childhood. Alternatively, children may only infer that all physical events have causes over the first 2 years of life as they acquire experience viewing causal events, and causing events to occur themselves.

We have argued that the toddlers in the current study inferred that there was a cause of the light’s flashing when it appeared to occur spontaneously and that they accepted the button as a plausible candidate cause. However, our results cannot tell us whether toddlers inferred that the button was *the* cause of the light’s flashing, or simply *a* potential cause of the light’s flashing. That is, toddlers in the Spontaneous condition may have treated the button as a potential cause of subsequent activations of the light without inferring that it caused the original light flash. Similarly, toddlers in the Spontaneous condition may not have inferred the presence of a hidden cause when they saw the light activate on its own, and nonetheless have been selectively motivated to attend to (Experiment 1) and explore (Experiments 2 and 3) the plausible cause when it became visible. Future research might try to look at the timing of children’s inferences to see whether they attribute causes as soon as they observe apparently spontaneous events (and before they are shown plausible candidate causes) or whether observing spontaneous events makes children selectively attentive to plausible candidate causes when they subsequently appear.

There are advantages and disadvantages to using artifacts to assess toddlers’ causal reasoning. Toddlers have a lot of experience with lights turning on and off as a result of switches or button. Thus, if there are any contexts in which toddlers expect events to have causes, it should be for functional artifacts intentionally designed to have causal mechanisms in place. If toddlers expect causes when artifacts change states, this expectation may not extend to other kinds of physical events, let alone to biological, psychological and social events. In Experiments 1–3 we also provided toddlers with a plausible, familiar candidate cause: a button. Whether toddlers accept and explore a wider array of candidate causes to account for otherwise unexplained events remains an area for further inquiry. However, focusing on artifacts allowed us to look at whether toddlers posit candidate causes for unexplained events when they have the strongest reason to do so.

The expectation that physical events have causes allows learners to posit the existence of unobserved causes whenever events appear to occur spontaneously. However, toddlers might also make a stronger assumption: they might assume that causes produce their effects deterministically (Laplace, 1814/1951; Schulz and Sommerville, 2006). A learner who makes this assumption can posit unobserved causes whenever events appear to occur stochastically: the learner may conclude either that a generative cause is sometimes missing or that an inhibitory cause is sometimes present. Some recent work in our lab suggests that toddlers may also make this inference (Wu et al., 2013) but further research remains necessary.

How toddlers acquire the inference that events have causes and constrain their hypothesis space in searching for candidate causes is an open question. Toddlers’ persistent exploration of the light box itself in the Spontaneous condition of Experiment 4 provides suggestive evidence that toddlers’ belief that unexplained events have causes might guide toddlers’ exploration and discovery of genuinely novel causal mechanisms. Given that physical contact has been shown to facilitate infants’ causal reasoning (Leslie, 1982; Cohen and Amsel, 1998) and appears to be a default expectation in children’s causal reasoning (Kushnir and Gopnik, 2007), the physical connection between the button and the light box likely also guided toddler’s causal search. Future work might look at how toddlers’ decisions about exploration trade-off the expectation that events have causes with inferences about the size search space, as well as how their expectations about spatial contact between causes and outcomes changes over development.

The current study suggests that toddlers will look for, intervene on, and explore plausible causes of otherwise apparently spontaneous events. This behavior may help support learning throughout development. If young children assume that physical events have causes, then seemingly spontaneous events can become a target for exploration and discovery, ultimately supporting the rich inquiry into non-obvious, unobserved, and even unobservable variables that characterizes human cognition.

## Conflict of Interest Statement

The authors declare that the research was conducted in the absence of any commercial or financial relationships that could be construed as a potential conflict of interest.
